# Photocatalytic activity of graphene oxide–TiO_2_ thin films sensitized by natural dyes extracted from *Bactris guineensis*

**DOI:** 10.1098/rsos.181824

**Published:** 2019-03-13

**Authors:** William Vallejo, Angie Rueda, Carlos Díaz-Uribe, Carlos Grande, Patricia Quintana

**Affiliations:** 1 Grupo de Fotoquímica y Fotobiología, Universidad del Atlántico, 081007 Puerto Colombia, Colombia; 2 Centro de Investigación y de Estudios Avanzados del IPN, CINVESTAV, Unidad Mérida, 97310 Mérida, YUC, México

**Keywords:** photocatalysis, TiO_2_, graphene oxide, sensitization, natural dyes

## Abstract

This study synthesized and characterized composites of graphene oxide and TiO_2_ (GO–TiO_2_). GO–TiO_2_ thin films were deposited using the doctor blade technique. Subsequently, the thin films were sensitized with a natural dye extracted from a Colombian source (*Bactris guineensis*). Thermogravimetric analysis, X-ray diffraction, Raman spectroscopy, scanning electron microscopy (SEM), X-ray photoelectron spectroscopy (XPS) and diffuse reflectance measurements were used for physico-chemical characterization. All the samples were polycrystalline in nature, and the diffraction signals corresponded to the TiO_2_ anatase crystalline phase. Raman spectroscopy and Fourier transform infrared spectroscopy (FTIR) verified the synthesis of composite thin films, and the SEM analysis confirmed the TiO_2_ films morphological modification after the process of GO incorporation and sensitization. XPS results suggested a possibility of appearance of titanium (III) through the formation of oxygen vacancies (O_v_). Furthermore, the optical results indicated that the presence of the natural sensitizer and GO improved the optical properties of TiO_2_ in the visible range. Finally, the photocatalytic degradation of methylene blue was studied under visible irradiation in aqueous solution, and pseudo-first-order model was used to obtain kinetic information about photocatalytic degradation. These results indicated that the presence of GO has an important synergistic effect in conjunction with the natural sensitizer, reaching a photocatalytic yield of 33%.

## 1. Introduction

In recent decades, the increased concentration of organic dyes in water has been a problem of growing concern; the textiles, paper and plastics industries use dyes in the manufacture of their products and consume a large quantity of water, increasing quantities of these pollutants in the final effluent [1,2]. Recent studies have demonstrated that heterogeneous photocatalysis is a promising technology as an alternative for water purification, especially in reducing the concentration of dyes in wastewater by the use of different kinds of semiconductors under a specific radiation source [3–5]. Nowadays, titanium dioxide (TiO_2_) has been broadly used as an efficient photocatalyst for environmental applications for both air and water purification due to its great quality/price ratio, chemical stability, good optical transparency and non-toxicity [5–7]. However, TiO_2_ has two drawbacks: (i) it is photocatalytically active under ultraviolet irradiation (*λ* < 350 nm) due to its high band gap energy value (3.2 eV) and (ii) low quantum efficiency in charge-carrier generation [8]. Many methods have been developed to increase or extend TiO_2_ photo-activity in the visible light region: (i) preparing micro and nanostructures (e.g. spheres and three-dimensional hierarchical) using different synthesis methods (e.g. CVD, CBD, solvothermal, sol–gel, template-free process and spray pyrolysis) [9–11], (ii) doping TiO_2_ structure with other atoms (e.g. Cu, Co, Ni, Cr, Mn, Mo, Nb, V, Fe, S, N, C, P and I) [12–14], (iii) surface plasmon resonance (e.g. Ag/TiO_2_, Au/TiO_2_ and Pt/TiO_2_) [15], (iv) coupled semiconductor (e.g. CaTe/TiO_2_, CdS/TiO_2_, Bi_2_WO_6_ and ZnS/TiO_2_) [16–18], and (e) dye sensitization. Among these, one of the methods most studied for TiO_2_ modification is dye sensitization [19]. Different types of dyes are reported as sensitizers of TiO_2_ such as ruthenium complex, chlorophyll derivatives, natural porphyrins and others for both energetic and photocatalytic applications [20,21]. In the last decade, natural dyes have become an important source of dyes; Yuvapragasam *et al*. [22] synthesized and sensitized TiO_2_ nanorods with natural dyes extracted from *Sesbania grandiflora* flowers, *Camellia sinensis* leaves and *Rubia tinctorum* roots for dye-sensitized solar cells (DSSC) fabrication. Sathyajothi *et al.* [23] reported an investigation of two types of pigments as natural photosensitizers of TiO_2_ in DSSC, reporting henna (efficiency 1.08%) and beetroot (efficiency 1.3%) for each extract. Despite all the applications of natural dyes in solar cell systems, their use is limited as sensitizers for water purification. Buddee *et al*. [24] sensitized TiO_2_ with curcumin natural extract for enhanced photodegradation of dyes under visible light. Additionally, Zyoud *et al*. [25] sensitized TiO_2_ particles with anthocyanin for photodegradation of methyl orange, showing a complete dye mineralization under solar simulator radiations. Recent study has reported the improvement of the photocatalytic activity of TiO_2_ using *Syzygium cumini* as a natural sensitizer [26]. In Colombia, there are varieties of plants whose chemical constituents could satisfy the requirements for photocatalytic applications as sensitizers, the species *Bactris guineensis* called ‘corozo’ is a wild palm, which grows in Central/South America and is an important source of anthocyanins [27]. The modification of semiconductors with electron-donating materials (e.g. graphene, graphene oxide (GO) and other carbon materials) is another approach to improving the catalyst efficiency. This kind of donating materials reduces the recombination rate of electron–hole pairs by increasing the charge-carrier mobility [28]. Graphene sheets, nanotubes and nanoparticles with a higher specific surface area and excellent electronic properties can be used as a photocatalytic support for TiO_2_ to improve the photocatalytic activity [29]. Gunnagol *et al*. [30] obtained TiO_2_–graphene nanocomposites to study the photocatalytic degradation of Rhodamine B under UV irradiation (degradation yield 98%) and under visible light irradiation (degradation yield 87.19%), and reported that this activity was reached due to the large surface area, providing a greater number of surface active sites in the materials. Yang *et al.* [31] successfully prepared TiO_2_/graphene porous composites for methylene blue photodegradation, compared composites results to TiO_2_ Degussa P25 and verified that the composites increased light-absorbing capacity accelerating the separation of electrons and holes, suppressing the charge recombination owing to graphene properties. Finally, Stengl *et al*. [32] obtained TiO_2_/graphene nanocomposites by thermal hydrolysis of suspension with graphene nano-sheets, and titania-peroxo complex provided a good photocatalytic activity in the decomposition of butane under UV and visible light.

The present study demonstrated photocatalytic properties for structured composites based on TiO_2_/GO/natural dye extract.

## 2. Material and methods

### 2.1. Natural dyes extraction

The samples of the fruit *B. guineensis* (CO) were collected in the municipality of El Banco in the Department of Magdalena, Colombia (geographical location, latitude: 9°00′03″ N, longitude: 73^o^58′28″ W to 25 m above sea level). In natural dye extraction, the fruit was placed inside a percolator with an ethanol : water mixture (1 : 3), and the sample was acidified with HCl. The percolation process remained recirculating for 3 days. After that, distillation performed under reduced pressure was carried out to obtain crude dry extract.

### 2.2. TO_2_/graphene oxide composites synthesis

The GO used in this study was synthesized using the modified Hummer's method. Detailed information about the preparation and characterization of the GO used in this study can be found in a recently published study [33,34]. TiO_2_–GO catalysts were prepared by the sol–gel method. Titanium (IV) tetraisopropoxide (TTIP) was added very slowly in a mixture of isopropyl alcohol in acidic medium and the corresponding concentration of the GO dispersion previously performed by ultrasound. After that, hydrolysis was applied in reflux equipment controlling the temperature. The precipitate was dried at 120°C overnight and then calcined in air at 500°C for 4–5 h [35]. Finally, solid TiO_2_ with GO 0.15, 0.26, 0.51 and 1.1 (w/w %) were obtained; the samples were named A-TiO_2_–GO, B-TiO_2_–GO, C-TiO_2_–GO and D-TiO_2_–GO, respectively.

### 2.3. TO_2_/GO composites sensitization

TiO_2_–GO powder was mixed and macerated with polyethylene glycol and isopropyl alcohol at acidic pH and vigorously stirred to form a fine suspension. After that, the thin films were deposited on a glass substrate using the doctor blade method, and the film thickness was measured using a Veeco Dektak 150 profilometer (6 µm of thickness). The thin films were heated at 500°C for 1 h. For natural dye sensitization of the TiO_2_–GO thin films, the coatings were immersed in a solution at pH 3 of the previously extracted dye. The adsorption process was carried out for 24 h in constant agitation; after that, the sensitized film was washed and dried at room temperature.

### 2.4. TO_2_/GO composites characterization

The thermal decomposition behaviour of the compounds was studied by thermogravimetric analysis (TGA Discovery) in a range of 30–900°C with a nitrogen atmosphere and a ramp of 10°C min^−1^. Studies on adsorption–desorption of H_2_ at high pressure at 77.48 K and up to 6000 kPa were carried out in a high-pressure gas adsorption device (BELSORP MAX-LP, BEL Japan, Inc.). The optical properties of the thin films were analysed by the diffuse reflectance technique using a deuterium–halogen source (Mod. AvaLight DH-S-BAL) and an AvaSpec-2048 optical fibre coupled to a bifurcated fibre (FCR-7UV100-2-1X25) with an AFH-Eye of Avantes at 45°. The measurements were normalized using a standard white material (Spectralon as a reflective material, which has 99% reflectivity in the range of 200 nm to 2.5 µm, Ocean Optics WS-1-SL). The surface reflectance spectra of all the samples were recorded in the visible range in several zones to obtain a representative value (the illumination point was 1 mm in diameter) [36]. The semiconductor surface modification with GO was monitored by Raman spectroscopy (Witec Alpha 300 Raman/AFM equipment) in a range of 50–3500 cm^−1^ with a length excitation laser of 488 nm and an integration time of 1.00485 s. Furthermore, in order to obtain the compounds' structural properties, an X-ray diffraction analysis was performed (Bruker D8 Advance diffractometer, Bruker AXS, Germany) using Cu–K*α* radiation (*λ* = 0.15406 nm) under a voltage of 34 kV and a current of 25 mA. The morphological properties (size, distribution and dispersion of the particles) were analysed by scanning electron microscopy (model JSM-7600F, Jeol Ltd, Tokyo, Japan) under an excitation energy of 5 and 1 kV and samples metalized with gold–palladium for 30 s. The elemental composition was analysed by X-ray scattering spectroscopy. Modified and unmodified thin films with different GO loads were analysed by X-ray photoelectron spectroscopy (XPS). Data were obtained using the Thermo Scientific™ K-Alpha™ X-ray Photoelectron Spectrometer (ThermoFisher Scientific, USA), with a hemispheric analyser with an X-ray source from KR Al (*hν*) 1486.6 eV using a vacuum of approximately 10^−7^ Pa. The Avantage V. 59902 software was used in the analysis of peaks in the XPS spectra; furthermore, the decomposition of the peaks was performed with Gaussian components after a Shirley background subtraction.

### 2.5. Photocatalytic behaviour

This study sought to establish the GO and natural sensitization effect on the photodegradation activity of TiO_2_ under visible light radiation. For this purpose, methylene blue was chosen as the pollutant model taking into account that since 2010, the International Organization for Standardization (ISO) published Standard 10678:2010 [37]. Visible light was used as energetic source to determine the real impact of modification on the TiO_2_ photocatalytic properties. The thin films were immersed in blue methylene solution (25 ppm was used as target solution), and prior to irradiation, the system was magnetically stirred in the dark for 1 h to ensure the equilibrium of dye adsorption–desorption on the thin film surface. The system was irradiated by a visible lamp with an emission during 100 min. The concentration of dye was determined through the spectrophotometric method (Thermo Scientific–Genesys 10S) using 665 nm as fixed wavelength, with a calibration curve (correlation coefficient *R* = 0.997) using the Lambert–Beer equation.

## 3. Results and discussion

### 3.1. Adsorption characterization

**Figure 1.**
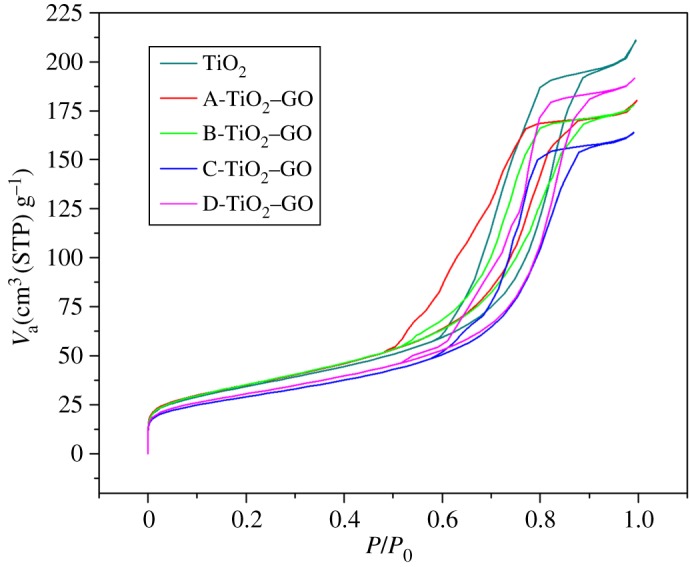
N_2_ adsorption and desorption isotherms for unmodified TiO_2_, A-TiO_2_–GO, B-TiO_2_–GO, C-TiO_2_–GO and D-TiO_2_–GO.

The surface area values and pore volume obtained from the method of Brunauer–Emmett–Teller (BET) and the adsorption and desorption isotherms are shown in figure 1. The specific surface area and total pore volume of the prepared composites are listed in table 1. The results of the BET analysis showed that composites had pore size around (8.06–9.23 nm), which are considered mesoporous composites according to IUPAC notation (mesoporous materials include the range of pore diameters between 2 and 50 nm); furthermore, the isotherms shown in figure 1 have the behaviour of type IV isotherms attributed to mesoporous solids [38]. Table 1 shows that, as GO concentration increases in the composite, the specific surface area decreases. According to data reported by Stengl *et al*. about the influence of the GO in the photocatalytic activity of TiO_2_, the reduction in the surface area could be due to an agglomeration given by the carbonaceous material, in which the GO is completely covered by semiconductor particles. Consequently, when it is required to measure the surface area of the modified materials, this depends mainly on the surface properties that present the anatase particles in the material [32].

**Table 1.** BET and total pore volume of the prepared samples.

catalysts	specific surface area (m^2^ g^−1^)	total pore volume (cm^3^ g^−1^)	pore size (nm)
unmodified TiO_2_	123.30	0.3229	9.23
A-TiO_2_–GO	126.32	0.2755	8.06
B-TiO_2_–GO	128.28	0.2746	7.05
C-TiO_2_–GO	104.33	0.2534	8.06
D-TiO_2_–GO	109.96	0.2956	9.13

### 3.2. TGA characterization

**Figure 2.**
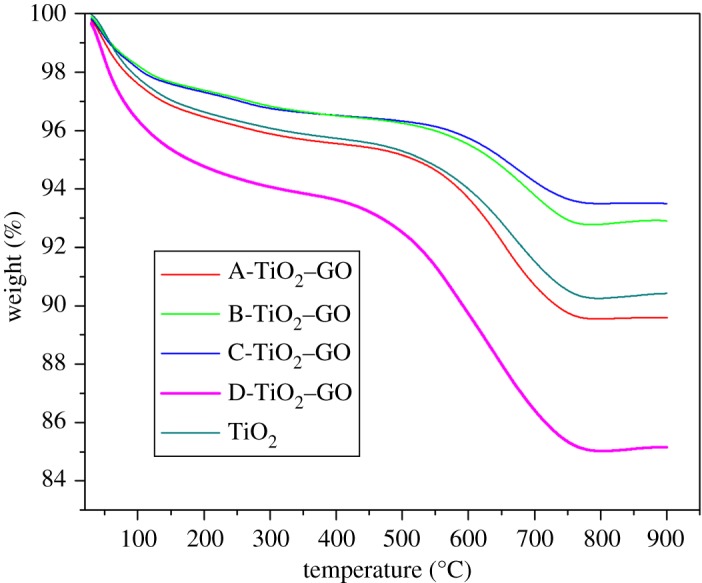
TGA curves for synthesized materials.

The thermal analysis and the stability of the catalysts were determined by TGA. Figure 2 shows the TGA curves for compounds. The unmodified TiO_2_ shows two stages of mass loss: (i) the first stage between 30 and 150°C with mass loss percentage of 2.96%—this change is associated with the evaporation of both water and solvent (isopropanol) molecules adsorbed on the surface of the material; (ii) second mass loss between 390 and 800°C with a loss percentage of 5.5%—this change is typical of the formation and reorganization of rutile phase crystalline structure (600–750°C); such results are in line with reports in the literature [39].

In relation to the modified samples (A-TiO_2_–GO, B-TiO_2_–GO, C-TiO_2_–GO and D-TiO_2_–GO), figure 2 shows three stages of mass loss: (i) the first stage is localized between 30 and 200°C with a loss percentage of 2.95%—this change is attributed to the evaporation of the water and solvent molecules present on the material surface [40]; (ii) the second stage is localized between 200 and 350°C with an average percentage of 0.74%—this mass loss corresponds to the removal of oxygen molecules contained in the labile functional groups (hydroxyl and carboxyl) present in the GO structure; at temperatures close to 350°C, the GO loses more stable oxygenated groups like carbonyls on the edge of GO sheets [41]; (iii) the last stage of change is localized between 450 and 800°C with a mass loss percentage of 4.14%—this stage is associated with the GO breakdown, the catalytic surface dehydroxylation and carbon substrate combustion (the carbon skeleton pyrolysis); furthermore, the formation and reorganization rutile phase occurs between 600 and 750°C [42]. For the sample D-TiO_2_–GO, two stages of weight loss were very resilient, with a loss percentage greater than the other three modified samples. This result can be attributed to the greater concentration of GO present in this sample; this greater proportion implies greater abundance of labile oxygen functional groups attached to the surface in its hexagonal structure, leading to greater mass losses in the second and third stages of the process [43].

### 3.3. Raman spectroscopy characterization

**Figure 3.**
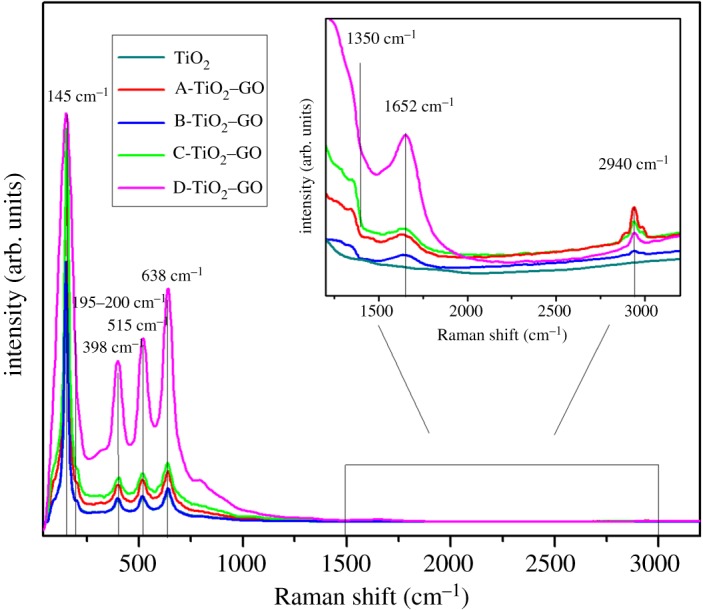
Raman spectra for the synthesized materials.

Figure 3 shows the Raman spectra of synthesized materials. All samples showed a similar pattern characteristic to the TiO_2_ anatase phase peak; the structure of the semiconductor anatase phase has six Raman active modes [44,45]:

3.1 $$\mathrm{anatase}\;=\;A_{1g}\;+\;2B_{1g}\;+\;3E_{g}.$$

The Raman spectrum shows a strong signal localized at 145 cm^−1^, which is caused by the external vibration of the TiO_2_ anatase structure. The peaks localized at 145, 195 and 638 cm^−1^ correspond to the vibrational modes *E*_1 g_, and the peak localized at 398 cm^−1^ can be attributed to the *B*_1 g_ vibration mode. Figure 3 shows the signal localized at 515 cm^−1^, which is a double-signal corresponding to the modes *A*_g_ and *B*_1 g_. Finally, none of the Raman bands corresponding to the TiO_2_ rutile phase were detected in the spectra [46–48]. For GO, two Raman active *E*_2 g_ modes are predicted, each doubly degenerate. Furthermore, in GO samples with defects, the overall momentum conservation can be satisfied by adding an electron-defect scattering event to the process, and two processes can be presented: (i) one-phonon defect-assisted process and (ii) two-phonon defect-assisted process [49]. Inside figure 3, a plot inset in the range of 1200–3500 cm^−1^ allows verifying the presence of the characteristic signals for GO, a band localized at 1350 cm^−1^ and another band localized at 1652 cm^−1^ corresponding to bands D and G, respectively. Band D is attributed to defects of sp^3^ carbons localized at the edges or in the plane of GO sheets, while band G is assigned to vibration of the atoms of carbon sp^2^ ordered in the hexagonal structure inside graphene [50,51]. Finally, the bands at 2940 cm^−1^ are associated with harmonics combination for bands D and D′, which takes place through a defect-induced triple resonance process involving both ‘inter-valley’ and ‘intra-valley’ scattering processes, whose intensity increases with the amount of disorder [52].

Furthermore, figure 3 shows that the TiO_2_ Raman signals enhanced after the GO content increase inside the catalysts (from TiO_2_ until D-TiO_2_–GO). Naumenko *et al*. [53] reported a significant electronic interaction between the TiO_2_ nanoparticles deposited by chemical vapour deposition on graphene sheets, and proposed that changes in Raman peak positions and intensity ratios could be due to the charge transfer process between graphene and TiO_2_ nanoparticles, and this increased the Raman signal of the TiO_2_ nanoparticles several times. Other reports have suggested that Raman intensity of TiO_2_ nanoparticles increased with the disorder of the graphene structure; this change in the intensities of the Raman peaks for increasing disorder has been reported for vacancies-type defects [54,55].

### 3.4. X-ray characterization

**Figure 4.**
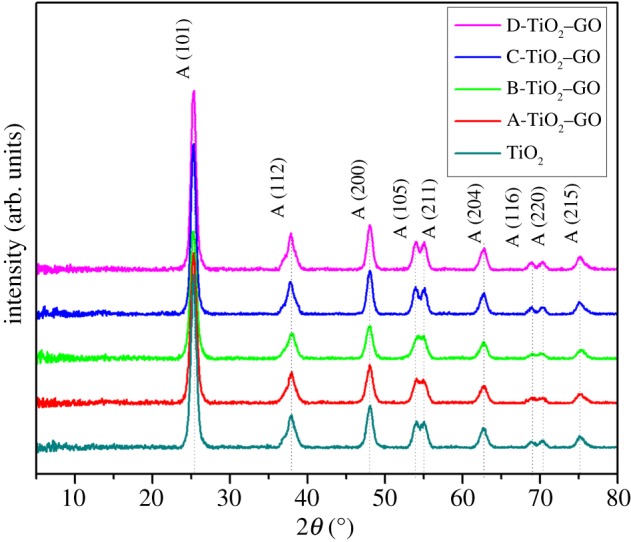
X-ray diffraction patterns of the composites synthesized in this study.

Figure 4 shows the X-ray diffraction patterns of the compounds. A qualitative analysis was carried out using the Diffract Suite Eva software (Bruker AXS, Germany) and the JCPDS database. The diffraction patterns indicate that films had a polycrystalline structure. Furthermore, the diffraction signals corresponded to the TiO_2_ anatase crystalline phase (JCPDS #021–1272). Figure 4 shows that the samples grown in a preferential crystalline plane were localized at 2*θ* = 25.5°; this signal corresponds to the plane (101). This result corroborates the information obtained from the Raman characterization.

Figure 4 shows that after the modification process, the diffraction pattern did not change significantly; in addition, none of the films showed any evidence of the presence of the rutile and brookite phases [56]. This result is in line with other reports, for instance, Rasoulnezhad *et al*. [57] did not report additional signals corresponding to the presence of doped elements into XRD patterns for TiO_2_ samples doped with S and Fe. The characteristic peak of GO cannot be appreciated due to the low concentration of GO used in the synthesis of the materials, which is in agreement with other reports on GO and TiO_2_ composites synthesis [58,59]. The crystalline domain size of the materials was calculated using the Debye–Scherrer formula and according to the preferential crystalline plane (101)

3.2 $$d_{hkl}=\frac{k\lambda }{\beta \cos(\theta )},$$

X-ray (Cu–K*α* radiation *λ* = 0.15406 nm), *β* is the diffraction angle to the full width at half maximum for the highest peak (101) and *θ* is the Scherrer diffraction angle [60], previous to applying the Debye–Scherrer formula, the instrumental broadening contribution was subtracted using DIFFRAC.SUITE EVA–XRD (Bruker, AXS, Germany). The average crystalline domain size obtained for all the TiO_2_–GO films was near 9.63 nm, while for the unmodified TiO_2_ thin films, it was 9.80 nm.

### 3.5. Sensitization process and FTIR characterization

**Figure 5.**
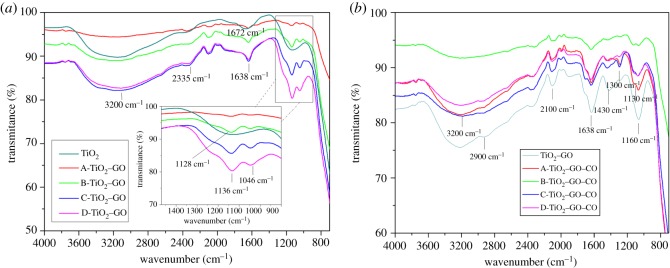
(*a*) FTIR spectra of the unmodified TiO_2_ and TiO_2_–GO photocatalysts. (*b*) FTIR spectra of TiO_2_ and TiO_2_–GO sensitized with natural extract.

After the synthesis and characterization of TiO_2_–GO thin films, the thin films were sensitized with natural extract. The FTIR characterization was carried out in the range of 700–4000 cm^−1^ to verify the type of chemical bonds present on thin films surface. Figure 5 shows the FTIR spectrum obtained for the unmodified and the modified TiO_2_ thin films.

Figure 5*a* shows a broad band in the range from 3500 to 3000 cm^−1^ and a band at 1672 cm^−1^, which are typical bands of both strain and bending of O–H bonds, and are attributed to the interaction of adsorbed water molecules on the thin films surface [61]. For TiO_2_–GO thin films, an intense wide band near 3200 cm^−1^ is observed, which increases in intensity as the concentration of GO increases in all the modified samples. This band could be attributed to the O–H stretching of the molecules of water absorbed on the catalyst surface and the hydroxyl groups present in the GO network—this behaviour was observed in all modified materials [62]. Furthermore, a weak band localized at 2335 cm^−1^ is assigned to O–C=O bonding, which are bonds present in the hexagonal GO. Another band is observed at 1638 cm^−1^, whose signal is assigned to the O–H bending of water molecules and C=C strain on the aromatic ring of GO. It is possible to observe a band at 1136 cm^−1^ which corresponds to the C–O bond of the epoxy groups, and a band at 1046 cm^−1^ can be assigned to =C–H bond stretching and C–OH strain for the alkoxy groups in the GO sheets [63]. Figure 5*b* shows the FTIR spectrum for the TiO_2_–GO films sensitized with anthocyanins extracted from corozo fruits (*B. guineensis*). The characteristic bands assigned to the O–H bond strain are observed. The bands localized at 2900, 2100 and 1638 cm^−1^ can be assigned to the vibrations of C–H bond, the C=O strain and the vibration of C=C conjugates of both the GO sheets and the anthocyanin structure of the natural dye, respectively. Figure 5*b* shows a typical band localized at 1430 cm^−1^ due to the OH–CH_2_ bonds of the phenolic rings present in the anthocyanins. The bands around 1300 and 1060 cm^−1^ are assigned to the asymmetric and symmetrical vibration of the C–O bond of the functional ether-type groups in the anthocyanins. This band is more intense for TiO_2_–sensitizers film than for TiO_2_–GO–sensitizer thin films, indicating a greater absorption of the natural dye on the semiconductor surface [64,65].

### 3.6. Morphological characterization

**Figure 6.**
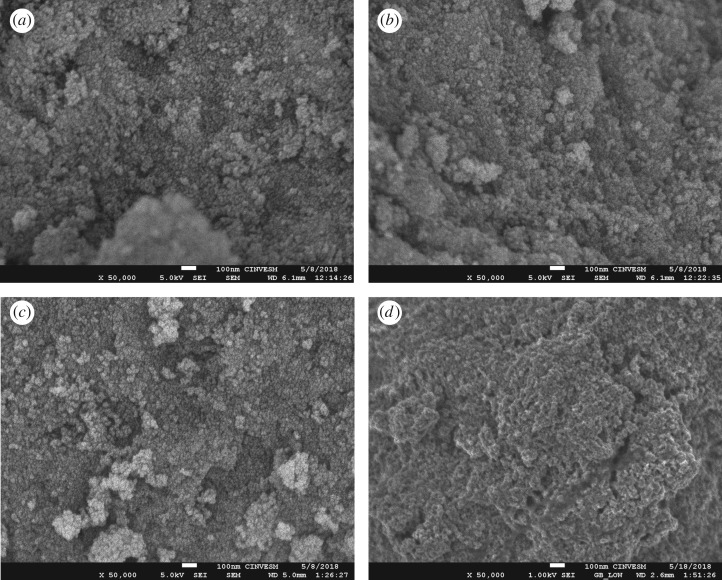
SEM images ×50 000: (*a*) TiO_2_, (*b*) A-TiO_2_–GO, (*c*) D-TiO_2_–GO and (*d*) D-TiO_2_–GO–CO.

Figure 6 shows SEM images with four TiO_2_ thin films. Figure 6*a* shows that the TiO_2_ films were formed by micro-aggregates with a narrow size margin of 15 nm. Figure 6*b,c* shows that, as the concentration of GO increased, agglomeration also increased. The agglomeration process is commonly reported when TiO_2_ nanoparticles are combined with graphene sheets. The agglomerates of TiO_2_ could enhance the photocatalytic activity because the photogenerated charge pairs can be efficiently separated through the inter-particle charge transfer within the agglomerates [66,67]. Furthermore, Ryu *et al*. [68] reported that rGO–TiO_2_ agglomerates promoted CH_3_CHO oxidation. Finally, figure 6*d* shows an agglomeration reduction in the particles on the surface after natural sensitization, owing to the fact that the natural dye adsorption on the catalyst surface improves the morphology of the films.

### 3.7. XPS thin films characterization

**Figure 7.**
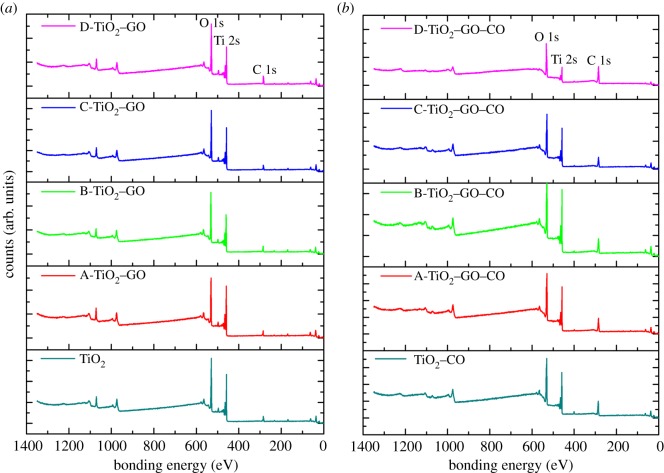
XPS spectrum of: (*a*) TiO_2_–GO thin films and (*b*) TiO_2_–GO thin films sensitized with anthocyanins extracted from the fruit of *B. guineensis*.

**Figure 8.**
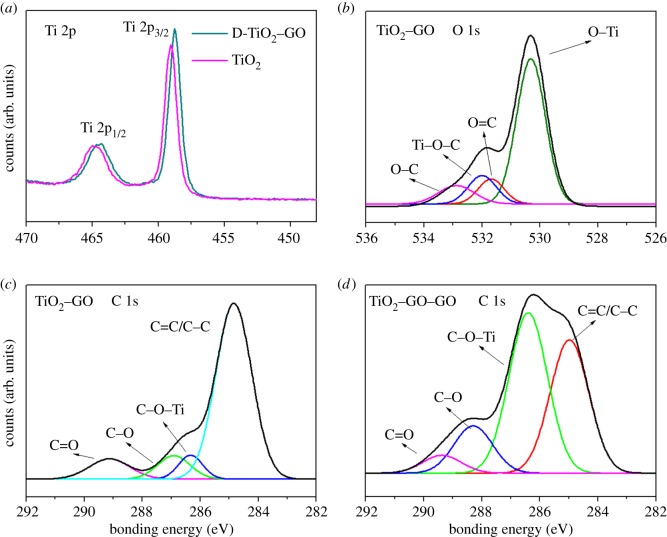
Peak decomposition of HRXPS to: (*a*) Ti 2p spectrum of TiO_2_ and D-TiO_2_–GO, (*b*) O 1s spectrum of D-TiO_2_–GO thin film, (*c*) C 1s spectrum of D-TiO_2_–GO and (*d*) C 1s spectrum of D-TiO_2_–GO–CO.

Figure 7 shows the XPS spectra obtained by all the synthesized films in the present study, and the spectra show the typical peaks for the elements C 1s, Ti 2p and O 1s corresponding to the bonding energy values localized at 284.5, 458 and 530 eV, respectively [69]. Figure 7*a* shows an increase in the signal for O 1s and C 1s as the concentration of GO increases in the samples, indicating a greater atomic percentage for these elements compared to the other samples—this is directly related to the concentration of graphene incorporated into the catalyst [70]. Figure 7*b* shows the spectra of the modified films with GO sensitized with anthocyanins extracted from *B. guineensis*, showing greater intensity in the peaks C 1s and O 1s, attributed to the extra contribution of these atoms by the anthocyanins from the fruit, which has an abundance of oxygen and carbon in its chemical structure, in addition to the contribution of GO [71]. In order to verify the kind of bonding in the synthesized compounds, a high-resolution XPS (HRXPS) was carried out in the specific region of (Ti 2p) for both TiO_2_ and D-TiO_2_–GO films (figure 8*a*). The peaks localized at 464.28 and 458.78 eV correspond to the spin-orbital splitting photoelectrons Ti 2p_1/2_ and Ti 2p_3/2_, respectively. The chemical shift of Ti 2p_1/2_ and Ti 2p_3/2_ was 5.6 eV, indicating that Ti^4+^ is present in the thin films [72]. Figure 8*a* also shows a red shift displacement of the spin to peak 0.6 eV, indicating the possibility of appearance of Ti^3+^ and that, under synthesis conditions, carbon tends to react with oxygen in the TiO_2_ lattice leading to the formation of oxygen vacancies (O_v_) and the low valence state of Ti^3+^ [73]. Rasoulnezhad *et al*. [74] reported the formation of Ti^3+^ species in N-doped TiO_2_ thin films deposited by chemical vapour deposition. The formation of O_v_ and Ti^3+^ could act as electron traps and inhibit the recombination process. Furthermore, the peak ascribed to Ti–C bonds at 281 eV was not detected in the TiO_2_–GO samples, which indicates that carbon was not doped into the TiO_2_ lattice [73,75,76]. The high-resolution spectra of O 1s and C 1s (figure 8*b,c*) were obtained from the sample with the highest load of GO (D-TiO_2_–GO); signals that were submitted to the decomposition process. Figure 8*b* shows the HRXPS spectrum to O 1s peak, with resolution of four signals: (i) the first is centred at 530.3 eV, attributed to the O–Ti bonding of the semiconductor lattice; (ii) the peak localized at 531.6 eV corresponds to the bonds O=C and −COO of the functional GO groups; (iii) the peak localized at 531.98 eV could be assigned to the Ti–O–C bond, a signal generated by the interaction between the oxygen atoms present in the functional groups in the graphene sheet and the TiO_2_ particles; (iv) the signal localized at 532.86 eV is assigned to the links C–OH and C–O–C [77,78]. Figure 8*c* shows HRXPS to element C 1s; the decomposition process of the signals shows resolution of four signals: (i) the main signal localized at 284.8 eV is assigned to carbon sp^2^ (C=C) and the unhybridized sp^3^ links (C–C); (ii) the second peak localized at 286.3 eV is attributed to C–O–Ti; this interaction is generated between the semiconductor and GO [79,80]. Finally, figure 8*d* shows HRXPS for the D-TiO_2_–GO film sensitized with natural extract, and in these spectra, the peak localized at 286.4 eV can be assigned to interaction C–O–Ti, indicating that the anchoring of the natural sensitizer to the TiO_2_ surface occurs through an interaction between semiconductor Ti and hydroxyl groups of the natural sensitizer anthocyanins; this is in line with computational studies reported on the adsorption of anthocyanins on TiO_2_ clusters [26].

### 3.8. Optical characterization

**Figure 9.**
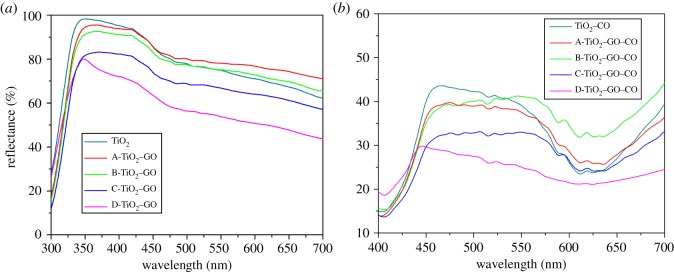
Reflectance diffuse spectra of: (*a*) TiO_2_–GO thin films and (*b*) TiO_2_–GO thin films sensitized with anthocyanins extracted from the fruit of *B. guineensis*.

Figure 9 shows the diffuse reflectance spectra for TiO_2_, TiO_2_–GO and TiO_2_–GO–CO thin films. The spectra show that GO affects significantly the optical properties of TiO_2_. Figure 9*a* shows a red shift of the absorption edge as GO concentration increases. The results suggest that the samples containing GO can absorb electromagnetic radiation with wavelength higher than TiO_2_; absorption increased linearly with the increase in GO concentration, which indicates that GO could narrow the band gap of TiO_2_ photocatalysts. For the samples sensitized with natural extract, a significant shift in the light absorption towards lengths greater than 400 nm was observed. The anthocyanins of the extract contained chromophore groups into their chemical structure, which absorb in this range of the electromagnetic spectrum. This result is in line with those in other reports [81–83].

The band gap energy value was determined for all samples using the Kubelka–Munk remission function [84]

3.3 $$\frac{k}{s}=F(R_{\infty })=\frac{{\left(1-R_{\infty }\right)}^{2}}{2R_{\infty }},$$

where $R_{\infty }$ is the material reflectance value and *F*($R_{\infty }$) represents the ratio between the absorption and the scattering coefficients *(k/s*), *F*($R_{\infty }$) is proportional to the constant of absorption of the material, an indication of the sample absorbance at a particular wavelength. From equation (3.3) and the curves shown in figure 9, an analogue to Tauc plots ((*F*($R_{\infty }$)*hv)^1/2^ against photon energy can be constructed, according to [85,86]

3.4 $${\left(F(R_{\infty })hv\right)}^{1/2}=A(hv-E_{g}).\;$$

**Figure 10.**
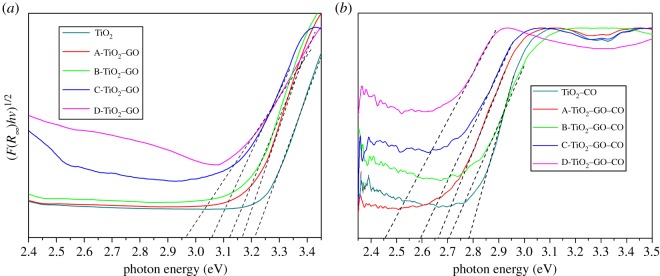
Kubelka–Munk plots and band gap energy estimation for: (*a*) TiO_2_–GO thin films and (*b*) TiO_2_–GO thin films sensitized with anthocyanins extracted from the fruit of *B. guineensis*.

Figure 10 shows plots of ${\left(F(R_{\infty })hv\right)}^{1/2}$ versus (*hv*) for the diffuse reflectance spectra shown in figure 9. The optical band gap of the films was determined by extrapolating the linear portion of the graph onto the *x*-axis [87]. Table 2 lists the optical properties of the thin films.

**Table 2.** Energy band gap values for the composites synthesized.

composites	band gap (eV)	composites	band gap (eV)
unmodified TiO_2_	3.21	TiO_2_–CO	2.78
A-TiO_2_–GO	3.16	A-TiO_2_–GO–CO	2.66
B-TiO_2_–GO	3.12	B-TiO_2_–GO–CO	2.70
C-TiO_2_–GO	3.05	C-TiO_2_–GO–CO	2.59
D-TiO_2_–GO	2.96	D-TiO_2_–GO–CO	2.45

Figure 10 shows that the unmodified TiO_2_ had a band gap value of 3.21 eV, which accords with reports in the literature [88,89]. The energy values for TiO_2_–GO were reduced as GO concentration was increased until reaching a value of 2.96 eV for the catalyst with the highest load of GO (D-TiO_2_–GO). The presence of GO allowed the generation of Ti^3+^ and O_v_, and such a low valence state could generate intra-gap states with lower energy than that in the TiO_2_ band gap, improving the absorption of light at higher wavelengths [73,75,90,91]. For the TiO_2_–GO–CO films, a greater change in the energy band gap values was observed; an energy value of 2.78 eV was obtained for TiO_2_–CO thin films up to 2.45 eV for D-TiO_2_–GO–CO thin films (thin film sensitized with higher loads of graphene). The presence of a natural sensitizer significantly improves the optical properties of TiO_2_–GO in the visible range of the electromagnetic spectrum, and this result is relevant to photocatalytic properties under visible irradiation [92].

### 3.9. Photocatalytic results

**Figure 11.**
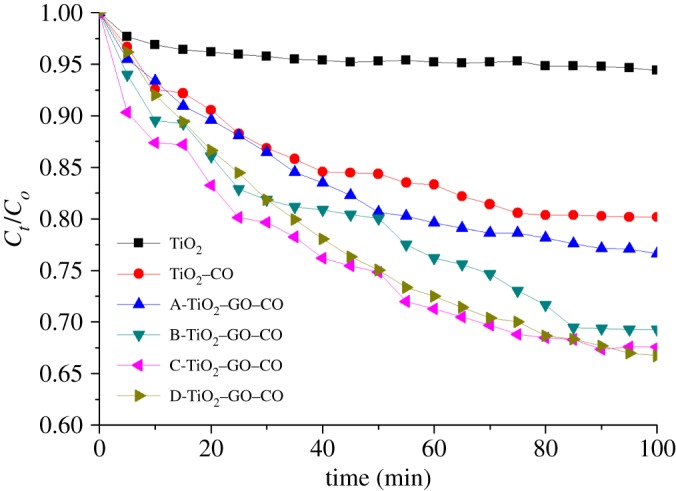
MB photocatalytic degradation by modified and sensitized TiO_2_–GO–CO materials after 100 min of visible irradiation.

The photocatalytic activity of TiO_2_–GO composites was studied by degradation of MB under visible light irradiation. TiO_2_, TiO_2_–CO and TiO_2_–GO–CO were compared with different GO loads. Figure 11 shows the results of photodegradation for different materials. All results indicate that modifications improved the catalytic activity of the semiconductor. Furthermore, the photodegradation yield increases when the concentration of GO increases inside TiO_2_ thin films. This behaviour is associated with the GO properties (e.g. electrical conductivity and capacity of charge transportation). GO sheets can promote the effective charge separation of electron–hole pair. In addition, the presence of Ti^3+^ and oxygen vacancies would facilitate the separation of charge carriers and suppress the recombination of charge carriers improving the photocatalytic activity [93,94].

Figure 11 shows that TiO_2_–GO–CO films showed greater photocatalytic activity than TiO_2_ films sensitized with natural extract (TiO_2_–CO), which indicates that the presence of GO has an important synergistic effect in conjunction with the natural sensitizer. The presence of GO can improve the electron transport transferred from the sensitizer to TiO_2_. Moreover, GO can reduce the recombination process of the photogenerated charge carrier; GO and sensitizer extend the photo-activity of TiO_2_ to the visible and improve the electrical transport significantly, increasing the values of photodegradation. The photodegradation kinetics of methylene blue (MB) was studied using the Langmuir–Hinshelwood kinetic model (L–H) [95,96]

3.5 $$v=-\frac{d[\mathrm{AM}]}{dt}=\frac{k\;*\;K[\mathrm{AM}]}{1+K[\mathrm{AM}]},$$

where *v* is the dye mineralization rate, *K* is the speed constant, [AM] is the concentration of methylene blue and *k* is the adsorption coefficient. Equation (3.5) can be explicitly resolved for (*t*) to use discrete changes in [AM] from the initial concentration to a zero-reference point. In the present case, an apparent first-order model can be supposed

3.6 $$v=-\frac{d[\mathrm{AM}]}{dt}=k_{\mathrm{app}}[\mathrm{AM}]=kK[\mathrm{AM}],$$

and

3.7 $$[\mathrm{AM}]={\left[\mathrm{AM}\right]}_{o}\;e^{-k_{\mathrm{app}}t}\;.$$

where time (*t*) is expressed in minutes and *k*_app_ (*k* * *K*) is the apparent reaction speed constant (min^−1^). The *k*_app_ values for synthesized catalysts are listed in table 3. Results show that the natural sensitization of TiO_2_ thin films increased the speed constant from a value of 3.40 × 10^−4^ min^−1^ for the unmodified TiO_2_ film to a value of 2.0 × 10^−3^ min^−1^ in TiO_2_–CO and, in this case, degradation yield increased by a factor of 4. The introduction of GO in the TiO_2_ semiconductor lattice was reflected in the increase in the speed constant from a value of 3.4 × 10^−4^ min^−1^ for the unmodified TiO_2_ film to a value of 3.9 × 10^−3^ min^−1^ for TiO_2_ modified with GO, which resulted from a reduction in the recombination process and the electrical conductivity of GO. In turn, for the sensitized TiO_2_–GO thin films, the *k*_app_ value reached higher values as GO concentration was increased in the films. The photodegradation rate was increased by 2 (D-TiO_2_–GO–CO thin films) in comparison to the TiO_2_ sensitized with natural extract, which indicates that the presence of GO has an important synergistic effect in conjunction with the natural sensitizer. Besides, the results suggest that the optical activity in visible range and the charge carrier's electrical transport were improved.

**Table 3.** MB degradation percentages and speed constant values for dye-sensitized TiO_2_–GO thin films.

thin films	*k*_app_ (min^−1^)	degradation (%)
TiO_2_	3.4 × 10^−4^	5.61
TiO_2_–CO	2.0 × 10^−3^	19.82
A-TiO_2_–GO–CO	2.4 × 10^−3^	23.38
B-TiO_2_–GO–CO	3.3 × 10^−3^	30.72
C-TiO_2_–GO–CO	3.5 × 10^−3^	32.42
D-TiO_2_–GO	3.9 × 10^−3^	33.28

Rasoulnezhad *et al*. [97] reported a *k*_app_ value of 7.3 × 10^−3^ min^−1^ for photocatalytic degradation of MB after 300 min under visible light irradiation using Fe-doped TiO_2_ thin films as photocatalyst. In another study, Yang *et al.* [98] reported a *k*_app_ value of 3.3 × 10^−3^ min^−1^ for photocatalytic degradation of MB after 180 min using poly-*o*-phenylenediamine-modified TiO_2_ nanocomposites as photocatalysts. Also, Jaihindh *et al*. [99] reported a *k*_app_ value of 8.7 × 10^−3^ min^−1^ for visible light photocatalytic degradation of MB after 150 min using GO-supported Ag-loaded Fe-doped TiO_2_ as photocatalysts. Sohail *et al.* [100] also reported a *k*_app_ value of 12.2 × 10^−3^ min^−1^ for photocatalytic degradation of MB after 120 min under UV light irradiation using TiO_2_-reduced GO nanoparticles as photocatalyst. In the present study, TiO_2_–GO–CO films have a suitable photocatalytic activity compared to these reports.

In order to build more knowledge in this research field, and taking into account the results presented in the previous section, a theoretical scheme of energetic levels was proposed for the material synthesized here (TiO_2_–GO–CO thin films). In the first stage, natural sensitizer (*S*) absorbs the visible light and, after that, it is excited to a state of greater energy, leaving an electron in the lowest energy unoccupied molecular orbital (LUMO) (equation (3.8)) [101]

3.8 $$S+hv_{\mathrm{visible}}\to \;\;S_{\mathrm{LUMO}\;}^{*}\;$$

and

3.9 $$S_{\mathrm{LUMO}\;}^{*}+\mathrm{Ti}O_{2}\to \;\;S_{\;}^{+}+\;\mathrm{Ti}O_{2}(e_{\mathrm{CB}\;}^{-}).$$

This electron can be transferred to the conduction band of TiO_2_ (equation (3.9)). At this point, Ti^3+^ and O_v_ act as electron traps and inhibit the recombination process (equations (3.10) and (3.11)). Furthermore, the photogenerated electron could be transferred to GO as well (equation (3.12)). The electrons localized in CB, Ti^3+^/O_v_ or GO can be transferred to an oxygen molecule to produce superoxide anion (equations (3.10)–(3.12)) [73,102]

3.10 $$e_{\mathrm{CB}\;}^{-}\to \;\;e^{-}(\;Ti^{3+})\;+\;O_{2}\to O_{2}^{∙-},$$

3.11 $$e_{\mathrm{CB}\;}^{-}\to \;\;e_{\;}^{-}(\;O_{v})\;+\;O_{2}\to O_{2}^{∙-}$$

3.12 $$\;\mathrm{and}\;e_{\mathrm{CB}\;}^{-}\to \;\;e^{-}(\;\mathrm{GO})+\;O_{2}\to O_{2}^{∙-}\;.$$

**Figure 12.**
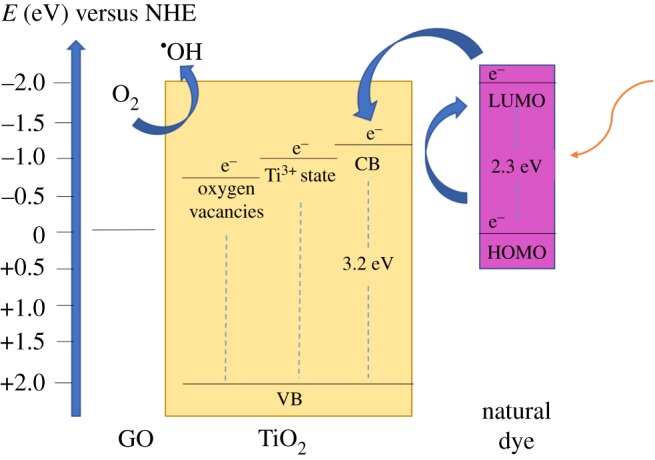
Scheme of energetic levels for TiO_2_–GO–CO thin films: energy band gap. Localized Ti^3+^ and O_V_ and HOMO-LUMO transition to natural dye [73].

In this stage, more reactive oxygen species can be generated and the degradation of pollutant begins [102,103] (figure 12)

3.13 $$O_{2}^{∙-}+\;H_{2}O\to OH^{∙}\;.$$

## 4. Conclusion

The present study synthesized TiO_2_–GO composites and studied the natural dye sensitization of TiO_2_–GO thin films with natural dyes extracted from *B. guineensis*. The spectroscopic, morphological and structural characterization of the composites was presented in detail. All results corroborated that the presence of GO and the natural sensitizer have an important synergistic effect on the physical chemistry properties of the composites. A red shift in the band gap values was detected after GO incorporation and natural dye sensitization from 3.21 eV (TiO_2_) to 2.45 eV (D-TiO_2_–GO). Results showed that the photodegradation yield of D-TiO_2_–GO was greater than that of TiO_2_–CO, indicating that GO could facilitate the separation of charge carriers suppressing the recombination of charge carriers, and the photodegradation yield was improved by the presence of GO and natural dye sensitization. The best photodegradation yield was reached by D-TiO_2_–GO, indicating that the presence of GO has an important synergistic effect in conjunction with the natural sensitizer. Finally, the results corroborate that natural sensitizers are an economic, harmless and promising source of dyes to be used in semiconductor sensitization.

## Data accessibility

Our data are deposited at the Dryad Digital Repository: http://dx.doi.org/10.5061/dryad.mc810bq [104].
